# The functional microbiome of arthropods

**DOI:** 10.1371/journal.pone.0176573

**Published:** 2017-05-05

**Authors:** Mauro Degli Esposti, Esperanza Martinez Romero

**Affiliations:** 1 Italian Institute of Technology, Genoa, Italy; 2 Center for Genomic Sciences, UNAM Campus of Cuernavaca, Cuernavaca, Morelos, Mexico; International Atomic Energy Agency, AUSTRIA

## Abstract

Many studies on the microbiome of animals have been reported but a comprehensive analysis is lacking. Here we present a meta-analysis on the microbiomes of arthropods and their terrestrial habitat, focusing on the functional profile of bacterial communities derived from metabolic traits that are essential for microbial life. We report a detailed analysis of probably the largest set of biochemically defined functional traits ever examined in microbiome studies. This work deals with the phylum proteobacteria, which is usually dominant in marine and terrestrial environments and covers all functions associated with microbiomes. The considerable variation in the distribution and abundance of proteobacteria in microbiomes has remained fundamentally unexplained. This analysis reveals discrete functional groups characteristic for adaptation to anaerobic conditions, which appear to be defined by environmental filtering of taxonomically related taxa. The biochemical diversification of the functional groups suggests an evolutionary trajectory in the structure of arthropods’ microbiome, from metabolically versatile to specialized proteobacterial organisms that are adapted to complex environments such as the gut of social insects. Bacterial distribution in arthropods’ microbiomes also shows taxonomic clusters that do not correspond to functional groups and may derive from other factors, including common contaminants of soil and reagents.

## Introduction

Genetic information on microbial communities is increasing dramatically due to the explosion of shotgun metagenomics [1–4]. The majority of this information regards the taxonomic composition of different microbiomes, while the functional significance of bacterial diversity and variation remains largely unknown [1,3,5]. Recent studies are beginning to bridge the information gap between taxonomy and function in microbiome research [3,5], revealing novel bacteria with metabolic traits that were not previously associated with the taxa into which they are classified [4,6]. For instance, metagenomic analysis of human gut microbiomes has revealed the genomes of uncultured alpha proteobacteria [4] that possess key genes for the anaerobic metabolism of eukaryotic organisms such as *Entamoeba* [6]. The discovery of such an important function in alpha proteobacteria [6] is remarkable because these bacteria normally form a minor component of the human gut microbiome, which is dominated by Firmicutes and Bacteroidetes [2]. However, alpha and gamma proteobacteria dominate the microbial communities of the oceans [3], including those associated with marine animals [7–9]. It is possible that anaerobic alpha proteobacteria such as *Azospirillum* sp. CAG:239 [4,6] may have an ancient terrestrial origin, given that its close relatives are predominant in composting microbiomes [10]. Terrestrial environments have been first colonized by arthropods, the most ancient representatives of which are millipedes and scorpions [11]. The gut microbiome of scorpions has been recently reported to contain a significant component of proteobacteria [12], many of which have been previously found in related arthropods such as ticks [13], as well as in cockroaches [14].

There is no comprehensive survey of the microbiomes of arthropods, which form the largest group of terrestrial animals and are adapted to all environments [11–18]. Here we present the first meta-analysis of the microbiome of arthropods, focusing on the major phylum of proteobacteria. The analysis combines, for the first time, a phylogenetically congruent taxonomic distribution of bacterial taxa with their functional profile, which has been derived from an in depth evaluation of several metabolic traits that are essential for microbial life. This evaluation encompassed probably the largest set of defined functional traits ever considered in microbiome or ecological studies. In the application to the microbiomes of arthropods, we found that discrete functional groups emerge from the distribution of taxa. Such functional groups appear to be phylogenetically conserved, but in part do not correspond to clusters of taxonomically related bacteria. The conserved pattern of metabolic traits across proteobacteria reveal common functions to thrive in arthropod guts and may also indicate stages in the evolution of the functional core in the microbiome of terrestrial animals.

## Results and discussion

### A novel visualization of bacterial distribution in the microbiome of arthropods

**Fig 1:**
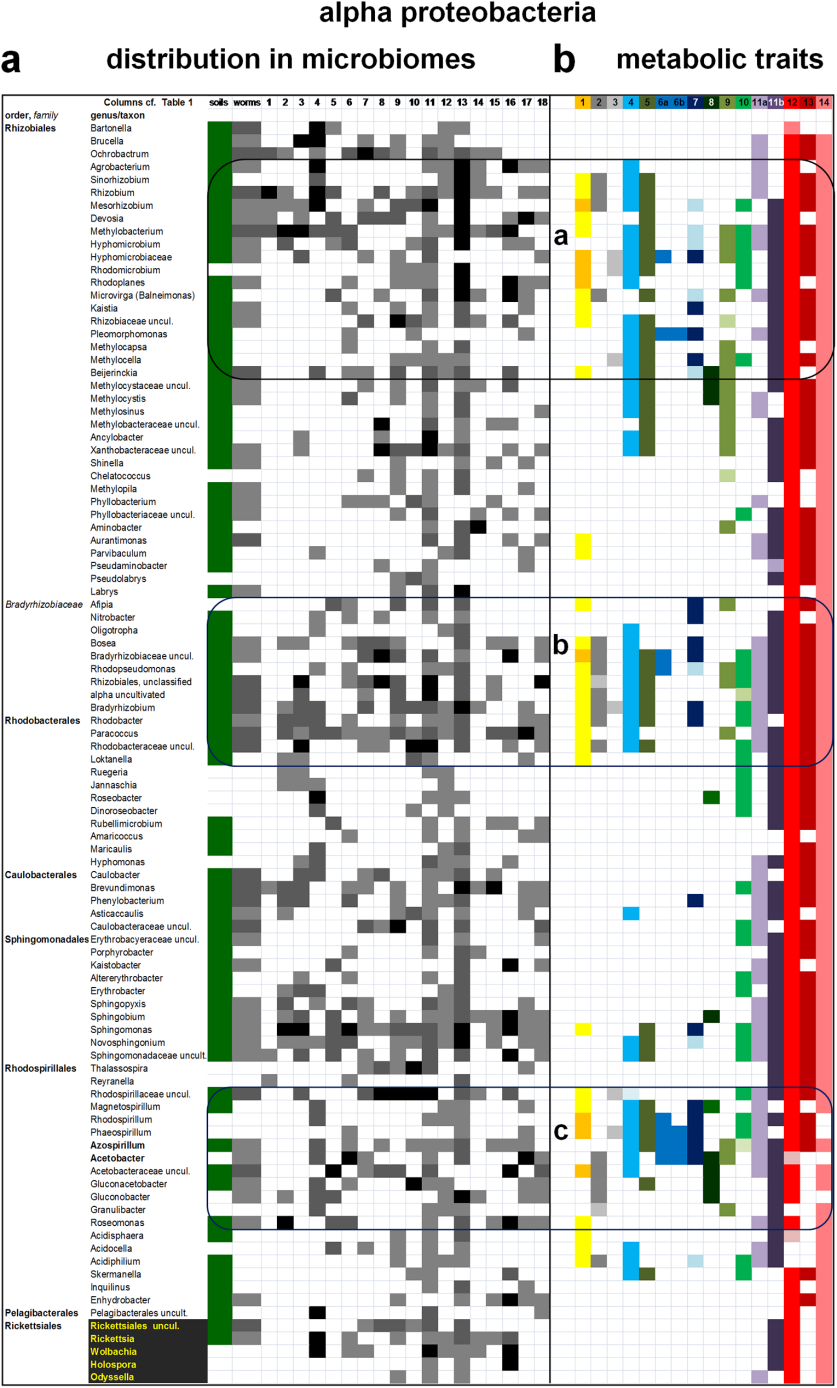
Visualization of the taxonomic distribution and metabolic traits of alpha proteobacteria in the microbiome of soils, *C*. *elegans* and 18 arthropods. Taxa of alpha proteobacteria found in the microbiome of soil [10,33,35], *C*.*elegans* [33,34] and 18 arthropods [12–14,16,17,19–32] (Table 1) were selected as described in the Methods and then organized in rows that followed their phylogenetic sequence from late to early branching. Taxa excluded because present only in two microbiomes or having few proteins sequenced are listed in S1 Table. **a.** The relative abundance of alpha proteobacterial taxa in the microbiome of soils and invertebrates was rendered in grayscale as described in the Methods, with most abundant organisms coloured in black. See S1 Table for quantitative details of bacterial abundance. The distribution of taxa in soil microbiome, however, was represented in green colour irrespective of abundance levels. Note the presence of clusters around nitrogen fixing Rhizobiales (top), Bradyrhizobiaceae plus some Rhodobacterales (middle) and within the orders of Sphingomonadales, Rhodospirillales and Rickettsiales (middle to bottom), in agreement with phenotypic information [73]. Rickettsiales are rendered in yellow over black background to underlie their nature of endocellular parasites. **b.** The 14 metabolic traits considered for the functional profile of microbiomes (in 16 columns, see Table 2 for details) were mapped on the phylogenetically organized distribution of taxa in part a. Their distribution segregates around bacteria that have been documented to possess MQ or RQ (rendered in orange) or potentially have MQ because they have key enzymes for its biosynthesis (rendered in yellow, see Methods). The resulting groups are labelled a (containing nitrogen fixing organisms, top), b (corresponding to the taxonomic cluster comprising Bradyrhizobiaceae and some Rhodobacterales in part a, middle) and c (confined to members of the order Rhodospirillales, bottom). These functional groups are boxed with solid lines that encompass the distribution of taxa in part a. Note that the taxonomic clusters within Sphingomonadales and Rickettsiales do not correspond to any group of metabolic traits.

**Fig 2:**
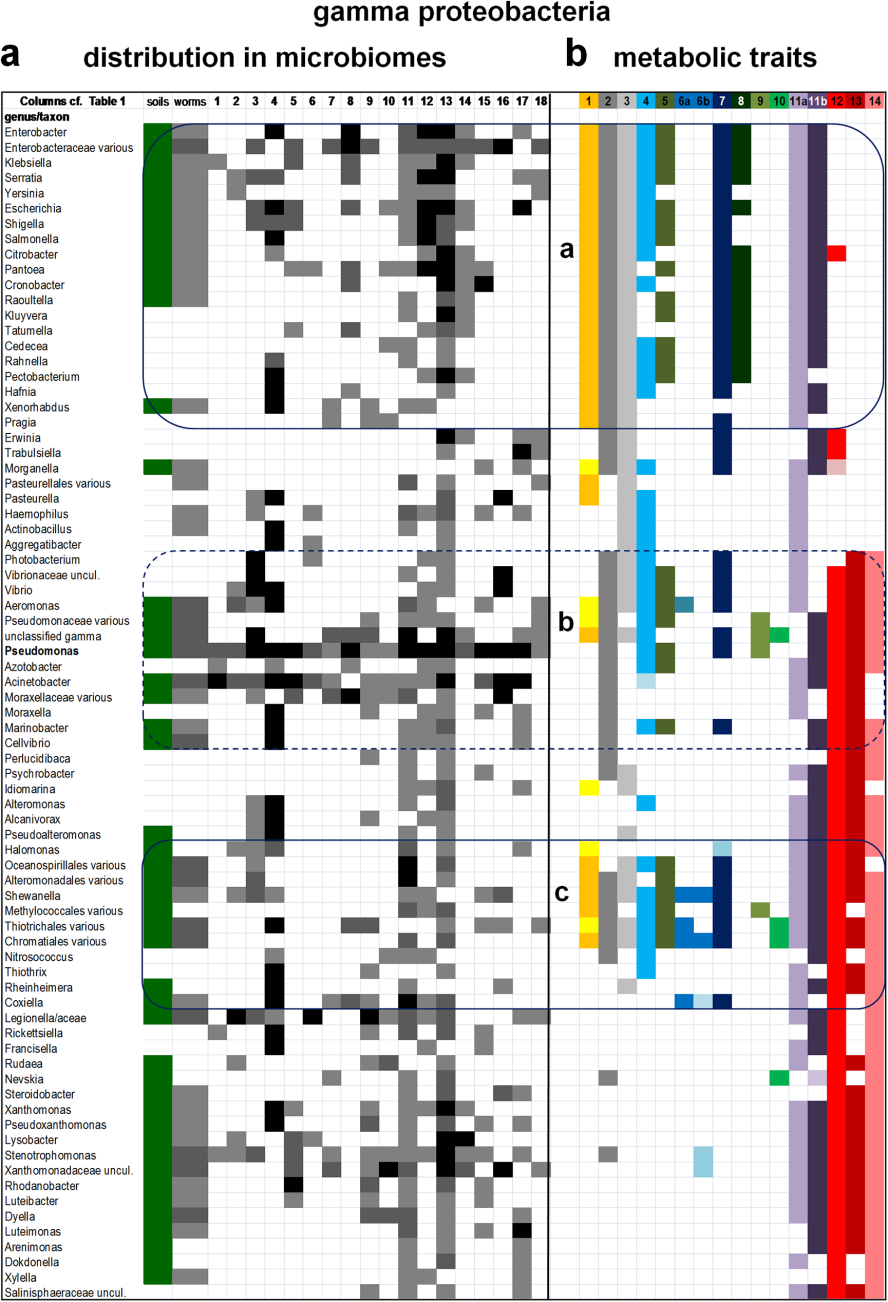
Visualization of the taxonomic distribution and metabolic traits of gamma proteobacteria in the microbiome of soils, *C*. *elegans* and 18 arthropods. Taxa of gamma proteobacteria in the microbiome of soils and invertebrates were organized as in Fig 1. a. The relative abundance of gamma proteobacterial taxa in each microbiome was rendered in grayscale as presented in Fig 1 and described in the Methods. See S1 Table for quantitative details of bacterial abundance. The distribution of taxa forms clear clusters encompassing most Enterobacteraceae (top) and combinations of organisms belonging to different orders: one including Pasteurellales, Vibrionales, Aeromonadales, Pseudomonadales and Cellvibrionales (middle), one spanning Oceanospirillales to Legionellales (middle to bottom) and one including Nevskiales and Xanthomonadales (bottom). Note that nearly one half of Enterobacterales found in arthropods are not present in soil and worm microbiomes, indicating a recent evolutionary diversification. b. Mapping of the14 metabolic traits considered for the functional profile of microbiomes (Table 2) segregates discrete groups as in alpha proteobacteria (Fig 1). These groups cluster around bacteria that have been documented or deduced to possess MQ and show other metabolic traits for anaerobic metabolism, in particular [FeFe]-hydrogenases. However, the separation between and functional group b (pivoting on Pseudomonadales, middle) and surrounding taxa is less sharp than in alpha proteobacteria (Fig 1) and consequently is boxed with dashed lines. In contrast, functional group c is well defined, despite its limited correspondences to taxonomic clusters in the distribution of bacteria (in part a). Note the complete absence of combined taxonomic traits in correspondence with the strong taxonomic cluster dominated by Xanthomonadales.

**Fig 3:**
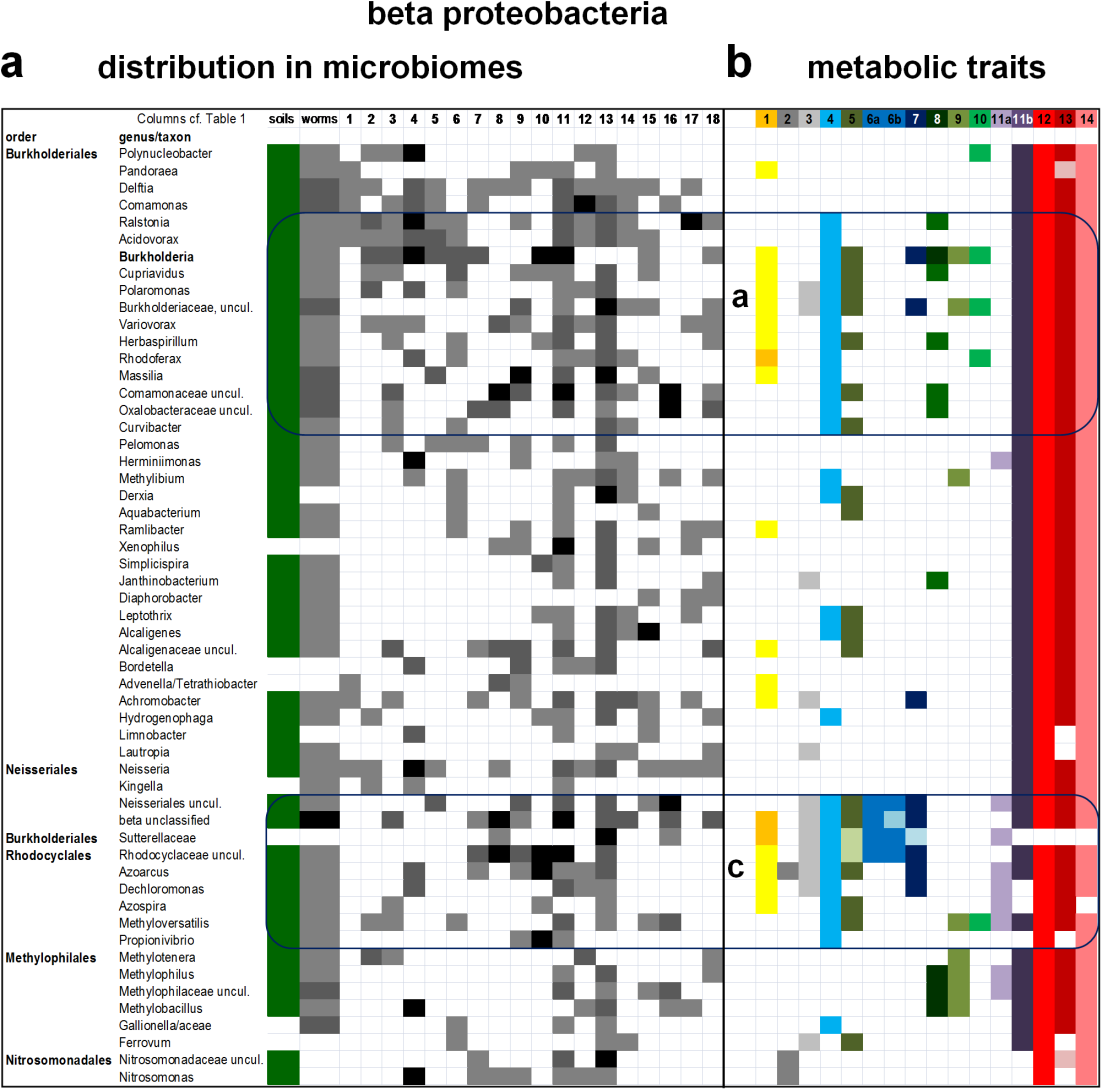
Visualization of the taxonomic distribution and metabolic traits of beta proteobacteria in the microbiome of soils, *C*. *elegans* and 18 arthropods. Taxa of beta proteobacteria in the microbiome of soils and invertebrates were organized as in Fig 1. **a.** The relative abundance of beta proteobacterial taxa in each microbiome was rendered in grayscale as presented in Fig 1 and described in the Methods. See S1 Table for quantitative details of bacterial abundance. The distribution of taxa forms various clusters from the top to the bottom of the phylogenetically organized sequence as for alpha (Fig 1) and gamma (Fig 2) proteobacteria. **b.** The mapping of the14 metabolic traits considered for the functional profile of microbiomes (Table 2) segregates two functional groups: group a (within the order of Burkholderiales, top) and group c (containing Sutterellaceae, some Neisseriales, unclassified beta and Rhodocyclales, bottom). These groups are well defined by statistically significant differences in Jaccard index and are rather similar to each other (see Fig 4C). Conversely, the scattered distribution of metabolic traits in the middle of the distribution did not allow the definition of another functional group for lack of statistically significant differences with surrounding taxa.

**Fig 4:**
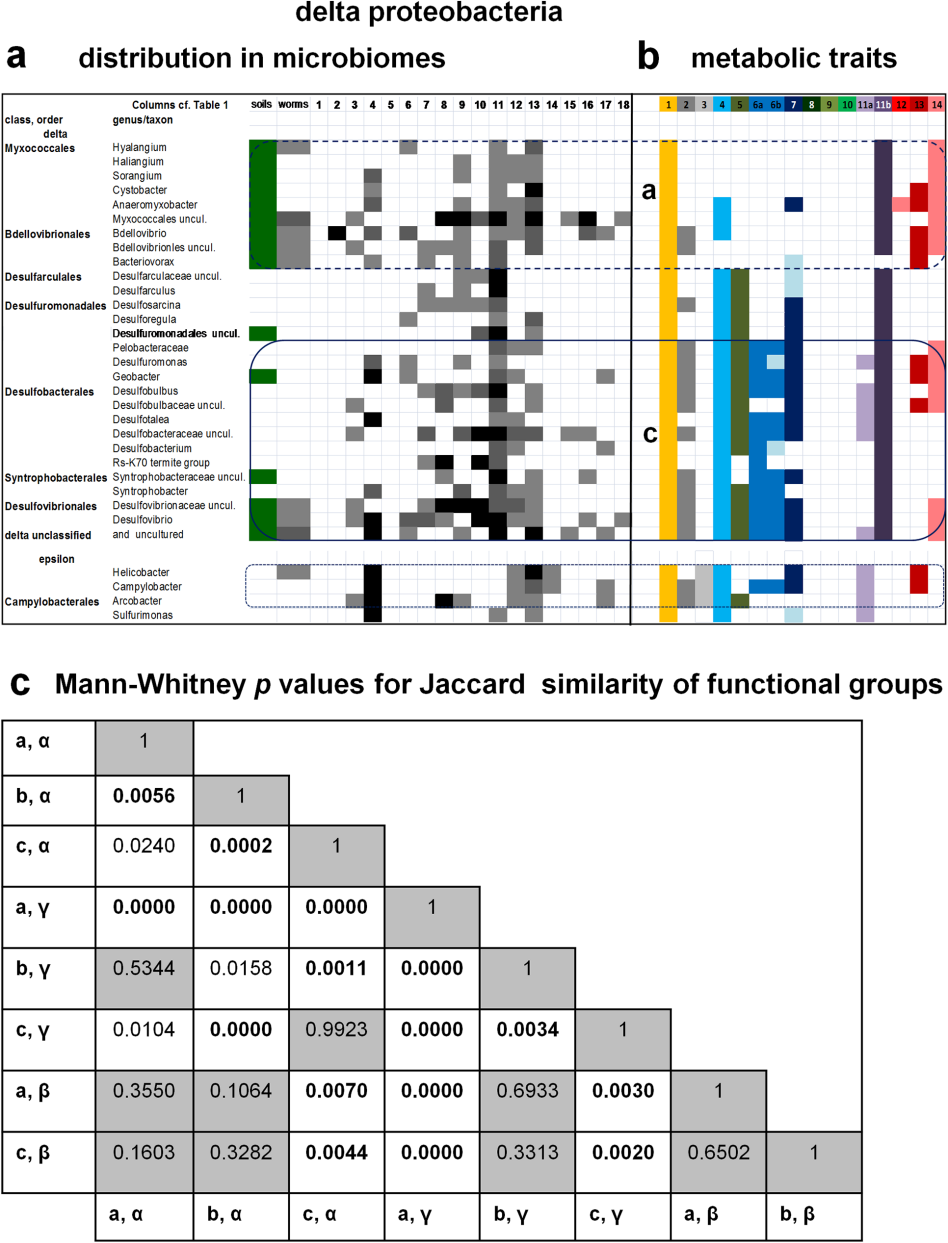
Visualization of the taxonomic distribution and metabolic traits of delta and epsilon proteobacteria and cross comparison of functional groups. Taxa of delta and epsilon proteobacteria in the microbiome of soils and invertebrates were organized as in Fig 1. **a.** The relative abundance of taxa in each microbiome was rendered in grayscale as presented in Fig 1 and described in the Methods. See S1 Table for quantitative details of bacterial abundance. The distribution of delta proteobacterial taxa shows clusters from the top to the bottom of the phylogenetically organized sequence, similarly to gamma proteobacteria (Fig 2). **b.** The major group of anaerobic, sulfate reducing delta proteobacteria defines the functional group boxed in the bottom. Aerobic predatory delta proteobacteria may form another functional group, which is not well separated from other taxa (top). Delta proteobacteria have an unusual succinate dehydrogenase enzyme, which is hybrid with fumarate reductase and lacks the membrane subunits that react with MQ. Therefore, it does not correspond to metabolic trait 3 of other proteobacteria and has been left empty in the diagram. Of note, the limited number of epsilon proteobacteria prevents the visualization of possible functional groups. **c.** Comparison of the functional groups of alpha, gamma, beta and delta proteobacteria was undertaken with the Jaccard index of similarity for 12 columns encompassing 11 anaerobic metabolic traits (see Methods and Table 2). The complete set of the Jaccard index values for each group (varying from 155 for group a of gamma proteobacteria to 28 for group a of beta proteobacteria) was compared using the non parametric test of Mann Whitney. Highly significant values of dissimilarity (*p* <0.01) are indicated in **bold**, while those largely non significant are highlighted in grey, indicating some similarity between the compared groups. The largest values for such as similarity have been found in groups a and b of beta and group a of alpha vs. group b of gamma proteobacteria, suggesting functional redundancy across taxa of different classes [3]. The functional group of deltaproteobacteria boxed in the bottom of part b was significantly different from all the other groups (*p* <0.002).

Several studies reporting microbiomes of arthropods [12–32] have been evaluated, extracting an assembly of nearly 500 taxa of proteobacteria, predominantly at the genus level of taxonomy but including also uncultured bacteria preliminarily classified at the family or order level (Table 1, Figs 1–4, S1 and S2 Tables).

**Table 1:** The table lists the microbiomes examined in this paper and the display items in which they appear.

column	Arthropods	Reference	method	Notes	genera/taxa*	display items
**1**	scorpions	12	16S rRNA & metagenomics	gut, two species	24	Figs 1–5, S1 S1 and S2 Tables.
**2**	prawns	17	metagenomics	aquatic (river), single species	48	Figs 1–5, S1 S1 and S2 Tables.
**3**	shrimps	23	16S rRNA	tiger shrimps, aquatic, wild and domesticated	102	Figs 1–5, S1 S1 and S2 Tables.
**4**	ticks	13	16S rRNA	ten species	141	Figs 1–5, S1 S1 and S2 Tables.
**5**	honeybee	22	16S rRNA	eggs and larvae	46	Figs 1–5, S1 S1 and S2 Tables.
**6**	fruitfly	19	16S rRNA	eggs and larvae	86	Figs 1–5, S1 S1 and S2 Tables.
**7**	cockroach	21	16S rRNA	gut, different diet, single species	70	Figs 1–5, S1 S1 and S2 Tables.
**8**	cockroaches	20	16S rRNA	gut, six species	65	Figs 1–5, S1 S1 and S2 Tables.
**9**	cockroaches	32	16S rRNA	gut, wood feeding, two species	82	Figs 1–5, S1 S1 and S2 Tables.
**10**	cockroaches	14	metagenomics	gut, two species	115	Figs 1–5, S1 S1 and S2 Tables.
**11**	termites	16	metagenomics	gut, six species	270	Figs 1–5, S1 S1 and S2 Tables.
**12**	ants	29	16S rRNA	leaf-cutting, two *Atta* species—workers and nest garden	120	Figs 1–5, S1 S1 and S2 Tables.
**13**	ants	26	16S rRNA	leaf-cutting—garden and nest, 35 samples	241	Figs 1–5, S1 S1 and S2 Tables.
**14**	ants	26	16S rRNA	leaf-cutting—workers only, seven samples	61	Figs 1–5, S1 S1 and S2 Tables.
**15**	lion ant	27	16S rRNA	whole body, larvae of a single species	67	Figs 1–5, S1 S1 and S2 Tables.
**16**	fleas	28	16S rRNA	16 specimen from two species	59	Figs 1–5, S1 S1 and S2 Tables.
**17**	beetles	24	16S rRNA	red palm, gut, two species	86	Figs 1–5, S1 S1 and S2 Tables.
**18**	beetles	31	16S rRNA	herbivore, gut, four species	77	Figs 1–5, S1 S1 and S2 Tables.
worms		33,34	16S rRNA & metagenomics	*C*.*elegans* induced microbiome, 27 samples	169	Figs 1–5, S1 S1 and S2 Tables.
soils		10,33,35	16S rRNA	*C*.*elegans* soils, 13 samples; rhizosphera of wild *Thymus*; poplar wood decomposting microbiome	225, 156, 22	Figs 1–5, S1 S1 and S2 Tables.
	isopods	30	16S rRNA	single species, gut and other organs	86	S1 and S2 Tables
contaminants		52,53	16S rRNA & metagenomics	different reagents and kits	70	Table 3 and S1 Table S1
oropharinx	humans	51	16S rRNA & metagenomics	oropharinx, healthy and affected humans	118	Table 3
HMP	humans	http://hmpdacc.org	16S rRNA & metagenomics	all tissues, healthy and affected humans	61	Table 3
gut	humans	2,4,50,76	metagenomics	gut, healthy and affected humans	22	Table 3

The column numbers refer to those of Figs 1 and 2.

*number of proteobacterial taxa after filtering undertaken as described in the Methods.

Cumulatively, these organisms represent one third of the proteobacterial genera currently listed in NCBI repositories (S3 Table). To effectively visualize the distribution of such a large set of organisms, proteobacterial taxa have been reduced to those present in at least three microbiomes of arthropods and arranged in a phylogenetic order of rows, descending from late to deep branching genera in each class (Fig 1 and S1 Table). This visualization significantly differs from the alphabetical order that is commonly used in listing bacterial taxa of microbiome assemblies [3,16,20,26–31], producing the first phylogenetically congruent distribution of bacterial organisms in animal and environmental microbiomes. The large microbiome of the model nematode, *C*. *elegans* [33,34], has been inserted in the distribution list of proteobacteria to provide a broad reference to terrestrial invertebrates (worms column in Fig 1A). The complementary data from soil samples of nematode worms [33] have been combined with those of other studies of soil microbiomes [10,35] and inserted at the beginning of the list of proteobacteria to frame their distribution within the microbial communities of terrestrial environments (soils column in Figs 1A, 2A, 3A and 4A).

Figs 1–4 show the distribution of alpha proteobacteria in the microbiome of soils, *C*.*elegans* and 18 arthropods. The complete set of organisms, including those excluded from the figures, is presented in S1 and S2 Tables. It shows even more clearly the taxonomic clustering of bacteria in three to five groups per proteobacterial class. Several organisms in the core of taxonomic clusters display high abundance levels (part a in Figs 1–3), but no clear pattern of taxonomic abundance could be discerned using standard approaches for evaluating beta diversity [36]. On the other hand, the majority of the proteobacterial taxa commonly found in the microbiomes of arthropods correspond to taxa that are present in soil microbiomes (part a in Figs 1–4), suggesting an environmental origin consistent with previous insights [5,10,12,14,16,18,26,32]. However, we note that the following taxa are found in arthropods but not in soils, or worms: many Rhodobacterales among alpha proteobacteria (Fig 1A); most Pasteurellales, Vibrionales and Alteromonadales among gamma proteobacteria (Fig 2A); and several non-predatory deltaproteobacteria (Fig 4A). Such alpha and gamma proteobacteria are predominantly marine or aquatic, forming taxonomic sub-clusters when their distribution is enlarged to the organisms present also in a single microbiome (S2 Table). Hence, their absence from soil microbiomes is presumably due to ecological restrains. Conversely, the delta proteobacterial taxa found in the microbiome of arthropods are very rarely seen in aquatic environments [3,7,8]. We discuss here the possible mechanisms of functional adaptation to the gut environment of arthropods.

### Metabolic traits define the functional profile of the microbiome of arthropods

To verify whether the taxonomic clusters of proteobacteria found in the microbiome of arthropods (e.g. Fig 1A) correlated with functional properties, we undertook an in depth analysis of the metabolic traits associated with the bacterial taxa examined. Specific metabolic traits shape the functional profile and ecological fitness of bacteria within different environments, both aquatic and terrestrial [1,2,5,7,15,18,29]. However, the appropriate definition of metabolic traits as meaningful functional characters depends upon the choice of the genomic elements that determine such traits and their conservation across the bacterial taxa that forms microbial communities [3,5,37–45]. Because the majority of metabolic pathways in proteobacteria are coded by operons, or by integrated genes from different genomic regions, we have associated a specific metabolic trait to a bacterial taxon when all the genetic elements that are essential for the function of the enzyme(s) defining that trait are present in the genome of at least one strain, species or OTU belonging to the same taxon [37,38]. This approach is biologically more meaningful than the common association of single genes to functional categories of either Cluster of Orthologous Genes (COG) or KEGG orthologous proteins [2,7,12,40,42] by using predefined databases such as PRICUSt [40], since operons often contain genes for proteins that are classified under different COG categories (Table 2, cf. [3,37]). We have considered about twenty different traits that can be associated with the bacterial taxa most frequently found in the microbiomes of arthropods and then have chosen a set of 14 metabolic traits that represent most pathways of energy conservation under micro-oxic and anaerobic conditions, which are usually present in the gut of arthropods and animals [2,6,16,18,31,37,41]. Indeed, fluctuating conditions of oxygen tension and niches of anaerobiosis are present in the guts of many arthropods, especially in social insects for which the most abundant taxonomic data are available [16,18,32,41,45]. Approximately one third of the traits we analyse here, for example nitrogen fixation, has been previously found to have a non random distribution across bacterial phyla [39] and are frequently present in proteobacteria forming the microbiomes of oceans [3]. At difference with previous studies, we have analysed an ample set of functionally related traits that follows a horizontal order coherent with biochemical and structural principles (see Methods).

**Table 2:** The table lists the metabolic traits considered in this work for the functional profile of microbiomes.

column	metabolic traits in bacterial genera	COG categories	Functional properties	References
**1**	menaquinone or rhodoquinone	E, H, I, M, Q, R	biosynthesis of low potential membrane quinones typical of anaerobic metabolism; weaker yellow when deduced from key metabolic enzymes for MQ biosynthesis	[46,49]
**2**	ancient complex I operon	C, P, R	including Nuo13 and green complex I, typical of anaerobic metabolism	[38,49]
**3**	fumarate reductase	C	reverse TCA cycle and reacting with MQ or RQ; often coupled to complex I	[38,67,68]
**4**	NiFe hydrogenase	C, K, O, P, R, S	group 1, 3 and 4 H2 consuming hydrogenases	[57]
**5**	nitrogenase—N2 fixation	C, E, O, P, R	catalytic and maturase subunits, associated with anaerobic metabolism	[38]
**6a**	[FeFe]-hydrogenase HydA	C	catalytic subunit, generally of M3 type, typical of anaerobic metabolism	[6,57]
**6b**	HydEFG maturases of [FeFe]-hydrogenase	C, O, R	assembly proteins for [FeFe]-hydrogenase activity, anaerobic metabolism	[6,57]
**7**	Pyruvate:Ferredoxin OxidoReductase, PFOR	C, E	energy conservation, coupled to [FeFe]-hydrogenase in anaerobic metabolism, in weaker blue when closely related enzyme are present instead	[6,38,57]
**8**	NADH-dependent nitrate assimilatory pathway	C, P, T	normally with nitrite reductase NirB fused wth NirD or deduced from the typical gene cluster for NADH-dependent assimilatory nitrate metabolism	[37,72]
**9**	methylotrophy	C, E, G, H	including methanotrophy an ammonia oxidation, requires oxygen, deduced also from the genomic presence of key methanol dehydrogenases	[37,71,72]
**10**	photosynthesis	C, E, G, H, K, O, P, Q, S	anoxygenic, reacting with (M)Q as electron acceptor	[69,70]
**11a**	*bd* type ubiquinol oxidase, bd-I	C, V	alternative oxidase bypassing cytochrome *c*	[37,38,62]
**11b**	*bd* type ubiquinol oxidase, CIO	C	alternative oxidase bypassing cytochrome *c*, low affinity for oxygen	[37,62]
**12**	cytochrome *bc*1 ubiquinol oxidoreductase	C	involved in aerobic metabolism and photosynthesis, reacting with Q and cytochrome *c*	[37,38]
**13**	*cbb*3 type cytochrome *c* oxidase	C, K, O, P, S	alternative oxidase with high affinity for oxygen reacting with cytochrome *c*	[37,38]
**14**	*aa*3 type cytochrome *c* oxidase	C, J, O	any of various COX operons for cytochrome *c*oxidase of aerobic metabolism, but sometimes with quinol oxidase activity too	[37,38]
	**other traits (continuous)**			
**15**	symbiosis	C, F, K, M, T	with either plants or animals	[5,38]
**16**	pathogenicity	D, E, K, O, P, U, V	to either plants or animals, including human opportunistic pathogens	[5,42,76–82]

The number and colour code of the columns is the same as those used in Fig 1.

### Discrete functional groups are present in the microbiome of arthropods

The mapping of the aforementioned metabolic traits has been integrated with the taxonomic distribution of the proteobacteria found in the microbiomes of arthropods using 16 colour-coded columns, as shown in part b of Figs 1–4. The taxonomic distribution of metabolic traits is clearly non random, in agreement with previous data [39]. For the phylogenetic groups of alpha proteobacteria shown in Fig 1, statistical analysis showed a *p* value of 6.7e^-14^ with the Friedman rank sum test for multiple correlated samples (in a two-way balanced complete block design) and a *p* value of 6.2e^-8^ with the Kruskal-Wallis test in their different distribution.

**Fig 5:**
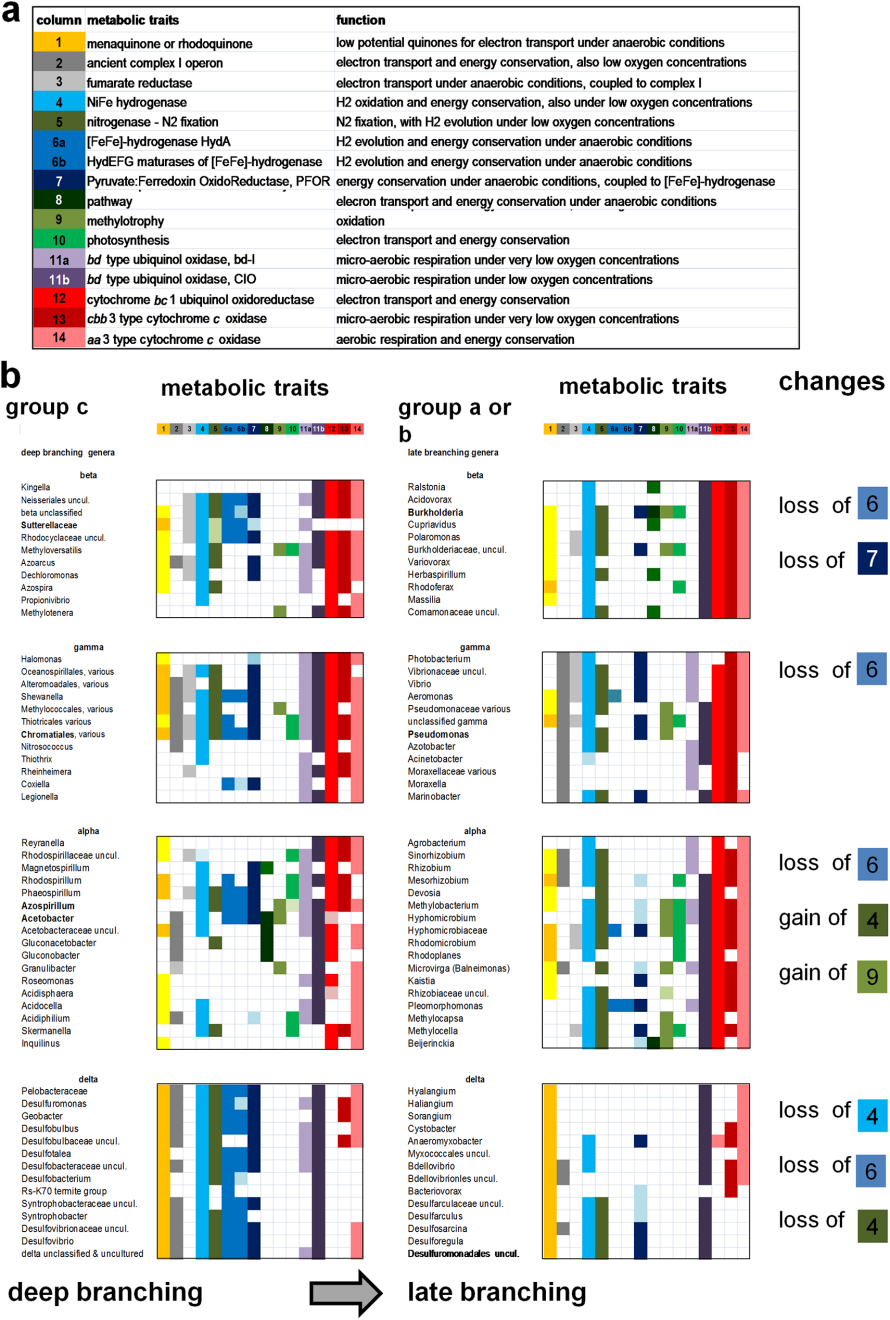
Comparison of discrete functional groups in diverse proteobacterial classes was rendered along evolutionary trajectories from late to early diverging taxa in both axes. **a.** Simplified definition of the metabolic traits considered here (cf. Table 2). **b.** The taxa that best define functional groups c (left) and a (right) were extracted from the combined distribution in delta (Fig 4), alpha (Fig 1), gamma (Fig 2) and beta proteobacteria (Fig 3) and organized following the evolutionary trajectory from early to late branching for both the proteobacterial classes (vertical axis) and the organisms within each class (horizontal axis). Note the similar clustering of metabolic traits in functional groups c (left), even if the statistical levels of Jaccard similarity were low (Fig 4C). Functional group a in gamma proteobacteria is not shown on the right of the illustration given its unique similarity properties (Figs 2 and 4C), and is substituted by functional group b of the same class. The metabolic traits that have been lost or gained with respect to functional groups c in each proteobacterial class are listed on the right, except for those reacting with cytochrome *c* (see text).

However, there is not a direct correspondence between the taxonomic clusters and the combinations of metabolic traits that accrue in functional groups on the basis of multivariate analysis. In alpha proteobacteria, for example, two functional groups near *Rhizobium* (group a, Fig 1 top) and Bradyrhizobiaceae (group b, Fig 1 middle) show highly significant difference in similarity indexes with respect to neighbour taxa. Conversely, the third functional group emerging from the functional profile of alpha proteobacteria, group c (Fig 1 bottom), is confined to taxa of the Rhodospirillales order. These include the genera possessing *Entamoeba*-like anaerobic metabolism [6], *Azospirillum* and *Acetobacter*, which show highly significant differences with respect to related Rhodospirillales such as *Acidisphaera* and *Inquilinus* that lie outside functional group c (*p* = 0.0007 with Mann Whitney test). A different situation is found in the functional profile of gamma proteobacteria, for their functional group c includes organisms from six different orders that do not seem to form a cohesive taxonomic cluster (Fig 2, bottom). Gamma proteobacteria frequently dominate the microbiome of arthropods [13,14,16,20,21,26–30] and include the ubiquitous genus *Pseudomonas* (Fig 2A and S1 Fig), which forms part of functional group b corresponding to a well defined taxonomic cluster (Fig 2, middle). Moreover, several members of the Enterobacteraceae family show a set of metabolic traits (labelled group a in Fig 2) characterized by the common presence of menaquinone (trait 1) and assimilatory nitrate reduction (trait 8), coupled with the absence of all systems reacting with cytochrome *c* (traits 12, 13 and 14, coloured in different shades of red, cf. Table 2). This functional group is unique to gamma proteobacteria since it does not show any significant similarity with other functional groups (Fig 4C). In contrast, group b of gamma proteobacteria is not significantly different from group a of alpha proteobacteria, as well as to the functional groups of beta proteobacteria (Fig 4C cf. Fig 3). The taxa from the latter class do not show a defined fictional group midway that present within the Burkholderiales order and that contributed by different deep branching taxa (functional groups a and c, respectively, in Fig 3B). The same appears to apply also to the functional profile of delta proteobacteria, which shows only a single defined functional group, labelled c in Fig 4 because it includes deep branching taxa as in group c of other proteobacterial classes (Fig 5).

The discrete functional groups we found in the bacteria of arthropods’ microbiomes pivot on taxa that have the documented presence of the low potential quinones, menaquinone and rhodoquinone (MQ and RQ, coloured in orange in Figs 1 and 2), or possess key enzymes for MQ biosynthesis [38,46–49]. The latter organisms are coloured in pale yellow in Figs 1–5. MQ is the central electron carrier of energy conserving systems under micro-aerobic and anaerobic conditions, receiving electrons from dehydrogenases oxidizing NADH (particularly complex I operons expressed under anaerobiosis [38,49]–trait 2 coloured in dark grey in Table 2 and Figs 1, 2 and 3), H_2_ and other low potential donors; it then transfers electrons to terminal oxidases of bd-type [37,38,41], as well as elements of nitrogen metabolism and fumarate reductase [37]. The latter enzyme (trait 3, coloured in light gray in Table 2 and Figs 1, 2 and 3) is widespread in facultatively anaerobic proteobacteria and together with complex I constitutes the simplest metabolic circuit for energy conservation under anaerobiosis. Fumarate reductase is also part of the reverse tricarboxylic acid cycle, which includes pyruvate:ferredoxin oxidoreductase (PFOR, coloured in dark blue in Table 2 and Figs 1, 2 and 3) and similar ferredoxin-reducing enzymes [38]. Reduced ferredoxin (or flavodoxin) that is produced by these enzymes is then re-oxidized by either nitrogenase [38] or [FeFe]-hydrogenase [6], enzymes that are structurally related to each other and yield the same product, H_2_ –hence their adjacent position in the series of metabolic traits considered here (Table 2 and Figs 1–4). H_2_ is re-oxidized by multiple forms of NiFe-hydrogenases (trait 5, coloured in pale blue in Table 2 and Figs 1, 2 and 3), which often are present in taxonomically related proteobacteria that have MQ or RQ (e.g. Fig 1). This indicates that a common network of energy metabolism pivoting on low potential quinones may constitute the functional core in the microbiome of arthropods.

Of interest, some functional groups are not significantly different from each other by multivariate analysis (Fig 4C). In particular, the functional groups of beta proteobacteria are not only similar to each other, but also statistically not dissimilar to group a of alpha and group b of alpha and gamma proteobacteria (Fig 4C), even if their visual comparison does shows some distinctive features (Fig 5B). Statistically significant levels of similarity between functional groups from different proteobacterial classes can be interpreted in terms of functional redundancy, a pattern previously found in diverse microbiomes [3,5,30,42,45]. Functional redundancy, together with the correspondence between functional groups and taxonomic clusters (for groups a, b and c in Fig 1, groups a and c in Fig 2 and group c in Fig 3), are consistent with the concept that environmental filtering could be the major driving force for structuring microbiomes [3,5,18,30]. To illustrate the functional redundancy across the taxonomic spectrum of bacterial microbiomes [3], the functional groups of all proteobacterial classes have been graphically compared from deep to late branching taxa along both orthogonal axes (Fig 5B). This representation shows a similar combination of metabolic traits for functional groups c, which are formed by deep branching taxa in each class (Fig 5B, left). It also indicates the progressive functional diversification of late branching taxa, which invariably have lost the anaerobic trait of [FeFe]-hydrogenases (Fig 5B, right). However, late branching alpha and gamma proteobacteria have also gained metabolic traits related to the nitrogen cycle and methylotrophy (Figs 1–4). Furthermore, potential complementarities between gamma and delta proteobacteria are apparent, since functional group a of the former (Enterobacterales) has lost the metabolic traits reacting with cytochrome *c* which have been gained by predatory organisms of the latter class (Figs 2 and 4). Consequently, both gene loss and gene acquisition contribute to the evolutionary changes of bacteria associated with the microbiome of arthropods, in accordance with previous findings and considerations [1,3,5,18,37,42,43,50,51].

Detailed comparison of the functional groups of proteobacteria in environmental and arthropods’ microbiomes suggests possible evolutionary scenarios for the acquisition of stable microbial communities in terrestrial animals. The earliest bacterial communities in the digestive system of arthropods could have derived from those naturally occurring in composting microbiomes of wood and soil [10,30,33,35], which are taxonomically related to the nest garden of leaf-cutting ants [10,26,29]. The nest garden environment of such social arthropods can be considered a natural bioreactor functioning as ‘external gut’ [26,29]; its large taxonomic breath matches that of termite guts (Figs 1 and 2, and S1 Table), which have the most complex microbial community of all arthropods [16,18]. The simple structure of the microbiome of scorpions [12] and sugar-feeding flying insects [18,19,22,30,41], as well as that of blood-feeding arthropods [13,28] and herbivore beetles [31], are clearly derived from their diverse diet that favours specific metabolic traits, for instance methylotrophy (trait 9, olive coloured in Table 2 and Figs 1–4). Methylotrophy here encompasses ammonia and methane oxidation (Table 2), previously considered as separate traits [3,39], and is concentrated within well defined functional groups of alpha and gamma proteobacteria (Figs 1 and 2) but is dispersed among beta proteobacteria (Fig 3), possibly reflecting different rates of evolution among proteobacterial classes. Conversely, the predominantly anaerobic delta and epsilon proteobacteria, which are scarcely present in soils and other terrestrial environments [10,30,33,35], are poorly represented in the microbiome of spiders [25] and isopods [30], and absent in the microbiome of scorpions [12]. The digestive apparatus of ancestral arthropods such as scorpions and spiders (aracnida [11]) is much simpler than that of cockroaches [14,21,32], termites [16,18] and beetles [18,24,31], which have specialized parts with little or no oxygen that would naturally favour the colonization by anaerobic delta and epsilon proteobacteria. Consequently, these proteobacteria would be late arrivals in the evolution of the microbiome of terrestrial animals, subsequently expanding their distribution in the anoxygenic intestinal tracts of mammals [2,4,9,50]. Bile salt-resistant *Bilophila* constitutes an example for this expansion of delta proteobacteria because it is common in human gut [2,50] but present only in one microbiome of arthropods (S2 Table).

### Constituents and contaminants of the microbiome of arthropods

The decoupling of some taxonomic clusters from functional groups in various classes of proteobacteria (Figs 1–4) has not been observed before in human [2,4,5,50,51] nor animal microbiomes [9,12,16], but echoes recent findings in sponges [42] and ocean microbiomes [3]. Because such a distribution is clearly non random, as verified by our statistical analysis and in accordance with a previous study [39], it could derive from ancestral founder lineages expanding into multiple related taxa after colonization of arthropods. This possibility appears to be plausible for the abundant groups of Sphingomonadales (Fig 1) and Xanthomonadales (Fig 2). Sphingomonadaceae, indeed, is the most represented proteobacterial family in the microbiomes of arthropods. However, Sphingomonadaceae are ubiquitous in soil, plant and aquatic environments, consequently constituting common contaminants in the kits and reagents used for DNA extraction and amplification [52,53] (Table 3).

**Table 3:** The table lists the 40 most common taxa found in the microbiomes of arthropods and their properties [74–84].

genus/taxa	column presence	common contaminant	ratio worms/soil#	in whitefly transcriptome*	in human oropharinx	in HMP, all tissues	functional group	NOTES and references
*Desulfovibrio*	**11**		1.25	yes			c, delta	environmental and in animal guts
Myxococcales, uncultured	**10**		0.01					predatory, environmental
*Bdellovibrio*	**9**		1.93		yes	yes		predatory, environmental
*Rhizobium*	**13**	yes	0.80	yes	yes		a, alpha	soil, plant symbiont, environmental
*Ochrobactrum*	**11**	yes	1.20	yes	yes		a, alpha	soil, environmental but also opportunistic pathogen [74]
*Methylobacterium*	**12**	yes	0.83	yes	yes		a, alpha	plant epiphyte, environmental but also amoeba resistant [75]
*Devosia*	**12**	yes	0.50		yes		a, alpha	soil and marine, environmental
*Mesorhizobium*	**9**	yes	0.08	yes	yes		a, alpha	soil, plant symbiont, environmental
Xanthobacteraceae, uncultured	**9**		n.a.		yes			environmental
*Rhodoplanes*	8		0.01				a, alpha	environmental and also amoeba resistant [75]
*Bradyrhizobium*	**11**	yes	0.03	yes	yes		b, alpha	soil, plant symbiont, environmental—but also amoeba resistant [75] and in human gut microbiota [76]
*Bosea*	**10**	yes	0.49				b, alpha	soil, environmental and also amoeba resistant [75]
*Brevundimonas*	**12**	yes	1.10		yes	yes		environmental but frequently present in insect and animal microbiomes
*Phenylobacterium*	**9**		0.42	yes				soil and aquatic, environmental—but also commensal of mouse and intracellular parasite of a human cell line
*Paracoccus*	**15**	yes	0.49	yes			b, alpha	soil, environmental
*Rhodobacter*	**10**	yes	0.19	yes	yes		b, alpha	soil, environmental
*Sphingomonas*	**15**	yes	0.22	yes	yes	yes		soil and marine/aquatic, environmental
*Sphingobium*	**13**	yes	0.28		yes	yes		soil and marine/aquatic, environmental
*Novosphingonium*	**13**	yes	0.50	yes	yes			environmental and opportunistic pathogen [77]
Sphingomonadaceae, unculured	**9**		n.a.					environmental and opportunistic human resident [78]
*Azospirillum*	**10**		**3.60**	yes		yes	c, alpha	soil, plant symbiont, environmental and also in human gut [4]
*Acetobacter*	7		n.a.	yes	yes	yes	c, alpha	commensal of sugar feeding insects, in beverages and human gut [4]
*Roseomonas*	**9**	yes	n.a.				c, alpha	environmental and opportunistic, especially in human skin [79]
Rhodospirillaceae, uncultured	**9**		0.03				c, alpha	mostly environmental
*Delftia*	**13**	yes	**4.00**	**yes**	yes	yes	a, beta	aquatic, environmental and in human gut [76]
*Ralstonia*	**13**	yes	0.37	yes	yes	yes	a, beta	soil and plant pathogen, environmental—but also opportunistic pathogen
*Acidovorax*	**11**	yes	0.79	yes	yes		a, beta	soil and plant pathogen, environmental
*Burkholderia*	**10**	yes	0.67	yes	yes	yes	a, beta	environmental, plant symbiont and human pathogen
*Comamonas*	**10**	yes	1.07	yes	yes			soil and compost, environmental—but also found in termite gut [80]
*Achromobacter*	**9**		**6.31**		yes			soil and aquatic, environmental—but also opportunistic pathogen [81]
beta unclassified & uncultured	**9**		1.11					mostly environmental
Enterobacteraceae, uncultured	**12**		**2.98**				a, gamma	predominantly resident in animal gut
*Escherichia*	**10**	yes	**6.04**	yes	yes	yes	a, gamma	human and animal gut resident; some strains are pathogenic
*Pantoea*	**9**		**3.11**	yes	yes	yes	a, gamma	plant seeds, environmental and opportunistic human pathogen
*Serratia*	**9**		**9.00**	**yes**			a, gamma	environmental but also animal gut resident and opportunistic human pathogen; one species is symbiont of aphids
*Pseudomonas*	**18**	yes	1.06	yes	yes	yes	b, gamma	environmental, including plant pathogens—but diffuse in animal and human microbiomes; frequently isolated in nosocomial infections and pathogenic
*Acinetobacter*	**15**	yes	0.44	yes	yes	yes	b, gamma	environmental but also opportunistic, frequently isolated in nosocomial infections and pathogenic—major antibiotic resistant infectious agent [82]
Moraxellaceae, uncultured	**9**	yes	n.a.	yes	yes	yes	b, gamma	in animal microbiota and opportunistic pathogens
*Legionella*	**11**		**9.48**	yes				endocellular parasite of amoebas and human pathogen, also environmental [83]
*Stenotrophomonas*	**13**	**yes**	**6.63**	**yes**	yes	yes		environmental and opportunistic human pathogen [84]; present in many animal microbiomes

The column presence refers to the number of microbiomes in which the taxon was found. Common contaminants were taken from Refs [52,53].

**#**calculated as described earlier [33] with some modifications, in **bold** when ca. 3-fold higher than unity; n.a., data not available.

*from unigene data of *B*.*tabaci* [15], in **bold** when very abundant.

The same applies to Xanthomonadales, ubiquitous *Pseudomonas* and other common proteobacteria found in the microbiome of arthropods (Table 3). This observation inevitably raises doubts on the genuine abundance and recurrence of such taxa in microbiomes, as discussed in a recent meta-analysis of the indoor environment [54]. Evidently, it is very hard to envisage to what extend the data presented in the studies examined here reflect possible contaminations, given that the majority of these studies did not produce negative controls with the reagents used. Moreover, most taxonomic data of the same studies derive from 16S rRNA sequences, which sometimes do not provide a good proxy for the metabolic versatility of closely related bacteria [18,39,43].

Nevertheless, the current analysis has identified a likely source of contamination in the marine proteobacteria that have been found in a single study (S2 Table), or form small taxonomic clusters within the microbiome of aquatic arthropods (Fig 1A, S1 Table). These organisms predominantly belong to the order of Rhodobacterales, Alteromonadales, Oceanospirillales and Vibrionales (S1 and S2 Tables), which together with Pelagibaterales are the most common bacteria in seawater [3,42]. On the other hand, the frequent presence of obligate intracellular parasites of the Rickettsiales and Legionellales orders [13,16,17,26,28,30] needs to be considered with caution, since these organisms can only be associated with microbiomes as parasites of amoebas or other metazoans. Strictly, therefore, they are not part of the community of bacterial gut symbionts and commensals, nor are environmental intruders. Indeed, their reduced genomes carry no distinctive functional trait, with the possible exception of some unclassified organisms of the *Coxiella* genus (Fig 2B, cf. [13,16]). To provide additional information for evaluating the problem of environmental contamination, we have calculated the average distribution ratio of proteobacteria between the experimental microbiome of worms and their soil environment (cf. Ref. [33]). Although distantly related to arthropods, *C*. *elegans* may be considered a reference invertebrate showing deterministic and reproducible structure in its microbiome [33,34]. Table 3 lists the values of the distribution ratio worms/soil for the forty most common proteobacteria in the microbiome of arthropods. Sphingomonadales invariably show low distribution values indicating lack of preference for *C*.*elegans*. The same applies to other bacteria that have been detected as common contaminants of kit and reagents [52,53], for example *Ochrobactrum* and *Brevundimonas* (Table 3). Notably, these organisms are not part of the functional groups found in alpha proteobacteria (Fig 1). However, equally low values of distribution ratio are associated with alpha proteobacteria that belong to discrete functional groups, for example *Methylobacterium*, *Bradyrhizobium* and *Paracoccus* (Table 3, cf. Fig 1), whereas high values of distribution ratio are associated with a few bacteria that do not belong to resolved functional groups (Table 3, cf. Fig 5). Hence, the experimental distribution ratio worms/soil is not well correlated with the functional profile of microbiomes and may reflect host selection processes specific to *C*. *elegans* [33]. It is interesting to note, anyway, that *Azospirillum* shows a high distribution ratio in worms and is not considered a common contaminant (Table 3 cf. [52,53]), despite its frequent presence in soil microbiomes [10,33].

The analysis of transcriptomes could provide a complementary approach to assess DNA contamination, for RNAs are normally easily degraded and therefore should not constitute significant contaminants in reagents and kits. One transcriptomics study on the white fly *Bemisia tabaci* [15] has reported a broad phylogenetic distribution of bacterial taxa that is comparable with the microbiomes examined here. *Delftia*, *Stenotrophomonas* and *Pseudomonas* were the most abundant proteobacterial organisms with gene transcripts in *Bemisia tabaci* (Table 3, cf. [15]). Such bacteria, therefore, could hardly be considered as contaminants in the microbiome of the white fly, despite their common presence in kits and reagents [52,53]. It remains to be seen whether the same applies to the microbiomes of other arthropods.

## Conclusion

This work presents the first meta-analysis of the microbiomes of arthropods, the dominant animals in terrestrial environments. It provides an informative visualization of the similarities and differences in the composition of microbiomes by combining taxa organized in phylogenetically congruent sequences with their functional profile, determined by a large set of metabolic traits coded by their genome (Figs 1–5). Such a presentation highlights taxonomic clusters corresponding to discrete functional groups that are conserved across proteobacterial classes (e.g. Fig 1). Notably, these groups would not be evident using the alphabetical listing of taxa that is regularly adopted in microbiome research following standard computer programs [3,16,26,31,36], as well as NCBI taxonomy (https://www.ncbi.nlm.nih.gov/taxonomy). They would also not be so evident by using principal component analysis or other statistical tools as in previous studies [2,4,54]. The presence of discrete functional groups with redundant metabolic capacity in all the microbiomes of arthropods (Figs 4C and 5, and S1 Fig) implies a strong effect of environmental (or habitat) filtering in shaping the structure of the bacterial community, in accordance with previous studies [3,5,18,30,36,54]. Conversely, the sharp, statistically significant distribution of taxa inside and outside such functional groups (Figs 1–4) would contradict the competitive lottery model for the structure of microbial communities [54,55], which therefore is unlikely to be fundamental in driving the microbiome of arthropods.

It is worth noting that our analysis encompasses probably the largest set of well defined traits that has ever been considered for evaluating the functional profile of animal microbiomes. In part, this analysis overlaps that previously reported for the ocean microbiome [3] and the functional profile of all bacteria [39], but it introduces the evaluation of low potential quinones and major bioenergetic enzymes reacting with them, which are instrumental to the adaptation to anaerobic conditions. Our analysis thus leads to insights into the possible functional evolution of animal microbiomes. In particular, the discrete functional groups of proteobacteria we have found in the microbiome of arthropods (Figs 1–5) may represent an ancestral core of the functional microbiome of terrestrial animals, subsequently expanded by inclusion of other Gram negative and Gram positive bacteria. The functional groups share metabolic traits of energy conservation that are fundamental for survival and fitness under micro-oxic and anaerobic conditions that were originally present in composting environmental communities [10,26,33], from which *Azospirillum* and other organisms are likely to have been filtered into animal microbiota. This initial group of metabolically versatile, facultatively anaerobic bacteria has remained associated with arthropods along their evolution and adaptation to terrestrial environments, progressively leading to more complex guts with low concentrations of oxygen [16,18,30,32,44]. Such complex gut environments have then favoured the colonization by anaerobic proteobacteria of the delta and epsilon class and the evolution of facultatively anaerobic specialists such as various Enterobacteraceae, together with the abundance of obligate anaerobes, especially Firmicutes [2]. Enterobacteraceae are among the most abundant organisms in the microbiome of nematodes [33,34] and arthropods [13,15,16,26,30], and have lost key metabolic traits of their gamma proteobacterial ancestors, while acquiring other pathways of energy metabolism, for instance NADH-dependent nitrate assimilation (Fig 2). They have also expanded additional traits that may favour their adaptation and competition within gut microbial communities, leading to pathological behaviour as well [2,4,39,50,55] (Table 3). The present analysis has also revealed taxonomic clusters that appear to be decoupled from functional groups and may have both biological and artefactual origins, the latter presumably due to common bacterial contaminants in the kits and reagents used for studying microbiomes [52–54]. Indeed, many of such contaminants correspond to the most frequent proteobacteria found in the microbiomes of arthropods and humans (Table 3). Other likely contaminants in the microbiome of terrestrial arthropods could be identified from their characteristic presence in seawater (cf. S1 and S2 Tables). Clearly, the approaches and considerations introduced in this work for proteobacteria can be extended to other bacterial phyla and the whole complexity of microbiomes. Even if the breath in the functional profile of bacterial communities will be expanded and refined, the anaerobic metabolism that has driven the microbiome structure in terrestrial animals is likely to remain fundamental as emerged from the present analysis.

## Methods

### Taxa from microbiomes and their phylogenetic organization

Primary data on the taxonomic composition of the microbiomes of arthropods have been obtained from Supporting Information files [13–32], as well as from the figures or tables of main articles [3,10,50] and direct communication with the Authors [12,35]. The taxonomic data of over twenty studies was organized in a master database, from which 18 different sets of taxa were selected for the final presentation (Table 1 and S1 Table) in combination with the functional profile (Figs 1–4). Studies were selected on the following criteria: i) presence of more than 20 different Operational Taxonomic Units (OTU) or bacterial species classified within proteobacteria for each arthropod species and ii) taxonomic diversity, i.e. taxa from more than one proteobacterial class, found under different ecological and experimental conditions, or in different tissue or structures. Results on *Wolbachia* or *Buchnera*, common insect endosymbionts within several invertebrates [18,25,30], were not considered. Data from detailed studies presenting thousand of bacterial taxa with associated statistics of reads [16,22,26,31,33] were filtered by applying threshold values, generally above 0.001% of the total reads for a given sample. Such a value was frequently found to remove all OTU of the Rickettsiales order, which should not be considered as *bona fide* members of a bacterial microbiome–the subject of this analysis—since they are obligate endocellular parasites of amoebas or metazoans, potentially deriving also from human contaminations. Table 1 lists the bacterial communities extracted from published works on the microbiome of arthropods and the cumulative number of taxa selected from each study, including the nest garden of leaf-cutting ants, which can be considered as the ‘external gut’ for these social organisms [26,29]. Indeed, the bacterial composition of nest garden communities of leaf-cutting ants (columns 12 and 13 in S1 Table, as well as in Figs 1–4) is similar to that in the hindgut of higher termites (column 11 in S1 Table, cf. [16,44]), which have the most complex bacterial community of all arthropods, approaching the complexity of mammalian gut microbiota [2,4,18,44,50]. The taxonomic classification reported in the original works has been maintained (Table 1) except for the genus *Enhydrobacter*, which has been listed among alpha Rhodospirillales as recommended [56] (S1 Table, see also Figs 1 and 2), instead of gamma proteobacteria [24,26,27,28,30–33]. The presence and abundance of proteobacterial taxa in diverse microbiomes of arthropods was organized in columns as in previous works, e.g. [42], but following an approximate phylogenetic sequence of the host organisms, from scorpions to beetles [11]. Ants and termites, which have the richest communities of proteobacteria [16,18,26,29,44], lie in the middle of such a sequence (Figs 1 and 2, and S1 Table). Relative abundance of taxa within each microbiome was rendered with a simplified heatmap having three grades of gray, while white symbolized absence. The lightest gray colour indicated the presence of a single OTU or strain for a given genus, or just its listing without specified read statistics if present in two or more different microbiomes. Black indicated the most abundant organisms, either because of documented high abundance of a single species per taxon or the presence of more than four strains or OTU per taxon in a given microbiome. Conversely, dark gray indicated intermediate abundance. The listing of microbiome taxa was organized in rows that followed as close as possible the sequence derived from detailed phylogenetic analysis, from late diverging down to deep branching organisms in each class of proteobacteria. The phylogenetic position of the various taxa was evaluated by combining relevant studies on the phylogenesis of alpha [57–59], gamma [60–62] and delta proteobacteria [63–65] with detailed analysis of conserved proteins carried out as described earlier [37,38,49,62,66]. The proteins include the catalytic subunit of aa3- and bd-type oxidases, which are not present in all proteobacteria [37,62], the NuoD and NuoL subunits of complex I [38,49], the catalytic subunit of [FeFe]-hydrogenase [6,57], cytochrome *b* of the bc1 complex [66], the flavoprotein subunit SdhA of succinate dehydrogenase and its related protein of fumarate reductase [67]. The phylogenetic position of some closely related beta proteobacteria was also adjusted on the basis of their functional profile, particularly in the case of the family Sutterellaceae, which contains anaerobic organisms predominantly found in gut microbiomes [4,48].

### Functional profile of proteobacterial microbiomes

The functional profile of the proteobacterial taxa organized in phylogenetic sequence as described above (S1 Table) was defined using 14 metabolic traits (listed in Table 2) that are fundamental for energy conservation under micro-oxic and anaerobic conditions [37,41,68]. These traits were defined by marker proteins for specific biochemical pathways, e.g. PFOR for substrate-level production of ATP using ferredoxin [6,68] (a metabolic trait often equated to fermentation), as well as complex metabolic phenotypes such as anoxygenic photosynthesis [39,69,70] and methylotrophy [71,72]. The latter was considered to include also methane and ammonia oxidation, reactions which are carried out by highly homologous enzymes [71] but are sometimes considered as separate metabolic traits in environmental studies [3,39,42]. Both methylotrophy (metabolic trait 9, Table 2) and photosynthesis (metabolic trait 10, Table 2) require the cooperation of different biochemical pathways coded by separate operons [69–72], which include gene products classified under different COG functional categories (Table 2). The same applies to the biosynthesis of MQ (metabolic trait 1, Table 2), the membrane electron carrier that links the following pathways of energy conservation essential to bacteria under anaerobic conditions [38,66]: ancient NADH dehydrogeases such as Nuo13 and green complex I [38,49] (metabolic trait 2, Table 2); fumarate reductase [67,68] (metabolic trait 3, Table 2); anoxygenic photosynthesis [69] (metabolic trait 10, Table 2); bd-type ubiquinol oxidase [38,62] (metabolic trait 11, separated in the bd-I type and Cyanide Insensitive Oxidase, CIO type, Table 2); and cytochrome bc1 complex [37,66] (metabolic trait 12, Table 2). Conversely, the soluble electron carrier ferredoxin links nitrogenase [38] (metabolic trait 5, Table 2), PFOR (metabolic trait 7, Table 2) and [FeFe]-hydrogenase [57,68] (metabolic trait 6), which is split in the catalytic subunit HydA (trait 6a, Table 2) and its maturase subunits HydEFG (trait 6b, Table 2) because they have separate and often independent genetic expression–the hydrogenase is functional only when these proteins are present together [6]. Other metabolic traits include group 1, 2 and 4 NiFe-hydrogenases that consume H_2_ [38,57] (trait 4, Table 2), NADH-dependent assimilatory nitrate reductase [37,72] (trait 8, Table 2) and the two terminal enzymes that oxidize cytochrome *c* [38]: cbb3-type oxidase (trait 13, Table 2) and aa3-type oxidases—in all their different operon types [37] (trait 14, Table 2). Of note, the presence or deduced biosynthesis of MQ has never been considered among the functional traits characterizing microbial communities [3,7,39,55], even if this low potential membrane quinone constitutes the central electron carrier in bacteria adapted to anaerobiosis, for example *Sutterella* [48]. Hence, the overall set of metabolic traits considered here is significantly larger than those published earlier, which may be functionally equivalent but are often combined with other traits that are either irrelevant to the microbiome environment of arthropods [3,39], or are superficially defined at the biochemical level. For instance, the most abundant functional trait found in ocean microbiomes has been defined as ‘aerobic chemoeterotrophy’ [3], which encompasses a wide range of chemoeterotrophic electron transport pathways, from nitrogen to iron, converging in a common set of terminal oxidases that may have different expression in related taxa (cf. [37,38]). We instead define such terminal oxidases as separate metabolic traits, since they are genetically independent and have very different evolutionary histories [37], while providing different levels of adaptation to low concentrations of oxygen [41]. However, the distribution of bo-type oxidases [37,41] is not presented here; after detailed analysis, it was discarded because these oxidases are too closely related to aa3-type oxidases [37]. The continuous complex traits of symbiosis and pathogenicity were also considered as in previous studies [3,55] (Table 2, bottom) but were excluded from the final functional profile because of their poor correlation with specific metabolic traits. They were instead considered among the defining features of the most common taxa in the microbiome of arthropods (Table 3). Previously considered metabolic pathways of the nitrogen and sulfur cycle [3,39,42] were not analyzed here because such pathways are equally involved in assimilatory and dissimilatory reactions, which cannot be precisely defined by genomic information alone [3,72]. Moreover, dissimilatory nitrate reduction often lacks key terminal enzymes when analyzed in animal microbiomes [42]. Complementary information on functional traits was taken from Bergey’s manual [73].

### Statistical analysis of data

Various approaches have been followed to evaluate beta diversity [36] and verify the statistical strength of the clustering of taxa and functional groups in the microbiome of arthropods. Given that checkerboard analysis by random permutation was not applicable to our data, because the rows of the distribution of taxa have been fixed by the abovementioned phylogenetic analysis, we undertook non parametric tests applied to sub-sections of the distribution tables after their conversion in binary matrices with presence (even partial) of a metabolic trait = 1 and absence = 0 (cf. Ref. [3,36,39]. In particular, local similarity in metabolic traits was evaluated across related taxa by using the Jaccard index calculated with the program PAST (https://folk.uio.no/ohammer/past) over segments of the binary distribution matrices including either 12 columns (encompassing 11 anaerobic metabolic traits, cf. Table 2) or the whole set of 14 metabolic traits, corresponding to 16 columns (cf. Table 2). Statistical analysis was routinely performed with non parametric tests using the programs MiniTab15 and PAST. Distinct functional groups were defined by values of Jaccard or Dice (Sorensen) index [36] that were significantly different (*p*≤ 0.01) than those of neighbour taxa, either closely related or distantly related within the same class. Comparison of pairs of functional groups within and between proteobacterial classes was undertaken by Mann Whitney tests of the combined Jaccard indexes of each group (see Fig 4C).

## Supporting information

S1 Fig Distribution of functional groups c in the microbiome of representative terrestrial arthropods. Each column representing the microbiome of specific arthropods as described in Table 1 (see also S1 Table) is split in two to visualize the distribution and abundance of the bacterial taxa (organized as in Fig 3 and rendered in black and gray for high and low abundance, respectively) and the cumulative number of taxa within functional group c in each proteobacterial class (in orange). Notably, all microbiomes contain at least one taxon forming part of functional group c in alpha and gamma proteobacteria.
(PDF)

S1 Table Distribution of proteobacterial taxa in the microbiome of arthropodes. The list is extended to the taxae that are present in at least two microbiomes of arthropods. The only exception is *Gilliamella*, because this anaerobic gamma proteobacterium dominates the gut microbiomes of bees [18,22]. The last column shows the taxa that have been reported to be common contaminants of kits and reagents [52,53]. The numbers OTU classified under a given taxon (generally at the genus level, but also at higher taxonomic levels) are indicated in each column; when rendered in bold, such numbers indicate high levels of relative abundance, after applying threshold values as described in the Methods section. Organisms highlighted in orange contain either RQ or MQ and are not listed in Figs 1–4. **%**, organism for which only a few protein sequences (generally below 200) are currently available in NCBI websites (https://www.ncbi.nlm.nih.gov/protein, accessed 26 November 2016).
(XLSX)

S2 Table Proteobacterial taxa present in a single microbiome of arthropods. Such taxad are thus excluded from S1 Table and Figs 1–4 and, generally, are not abundant in the microbiomes; their colour code is the same as that used in S1 Table. The last column on the right reports the presence of MQ.
(XLSX)

S3 Table General statistics of the proteobacterial taxa examined in this work. (PDF)
