# Supplementary figures and images for: The functional microbiome of arthropods
Source: PLoS One. 2017 May 5;12(5):e0176573. doi: 10.1371/journal.pone.0176573 (PMC5419562; doi:10.1371/journal.pone.0176573)

S1 Fig

microbiome of terrestrial arthropods

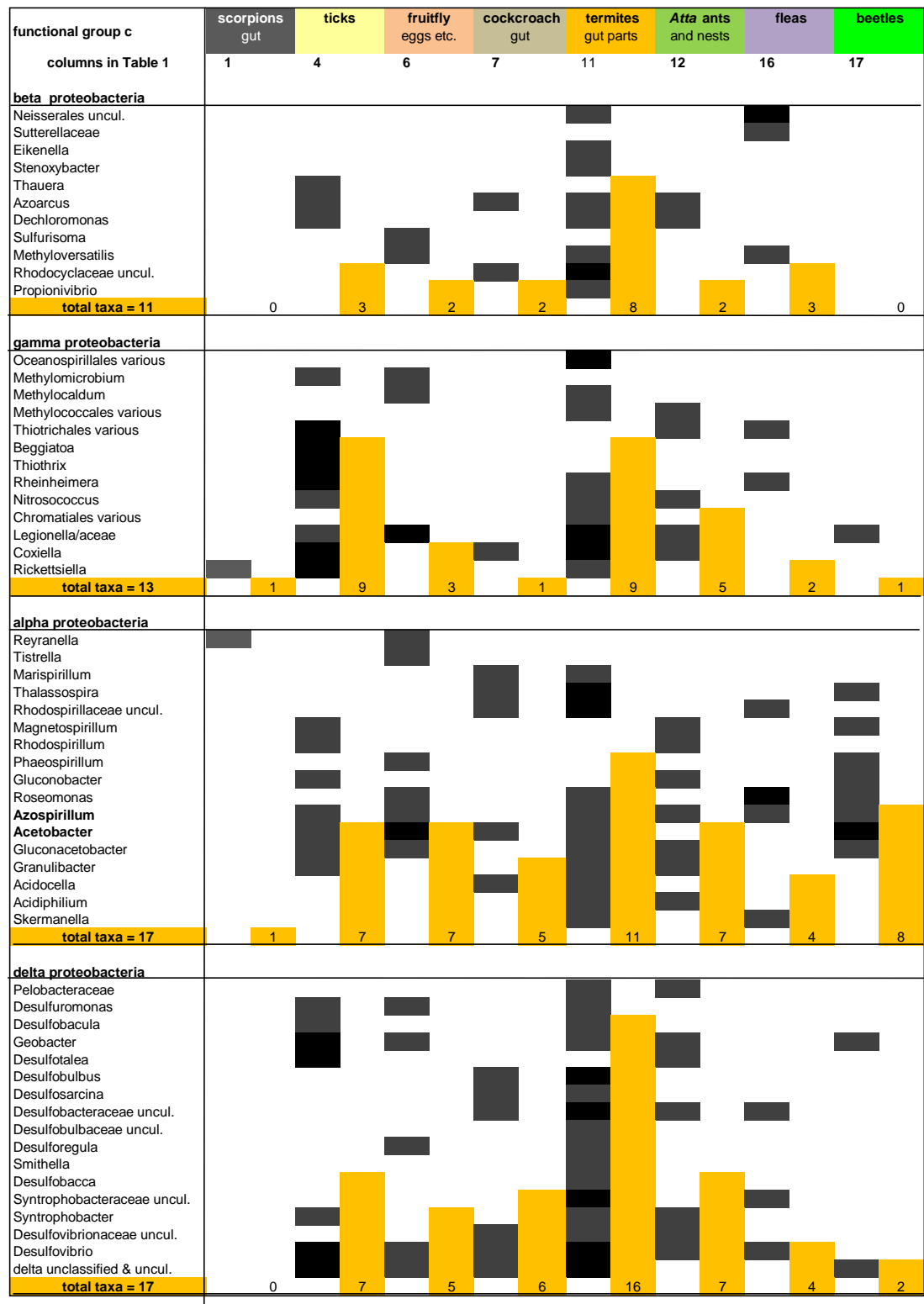

Supplement: S1 Fig — Each column representing the microbiome of specific arthropods as described in Table 1 (see also S1 Table) is split in two to visualize the distribution and abundance of the bacterial taxa (organized as in Fig 3 and rendered in black and gray for high and low abundance, respectively) and the cumulative number of taxa within functional group c in each proteobacterial class (in orange). Notably, all microbiomes contain at least one taxon forming part of functional group c in alpha and gamma proteobacteria. (PDF) [file pone.0176573.s001.pdf]
