# Supplementary material for: The functional microbiome of arthropods
Source: PLoS One. 2017 May 5;12(5):e0176573. doi: 10.1371/journal.pone.0176573 (PMC5419562; doi:10.1371/journal.pone.0176573)
Supplement: S3 Table — (PDF) [file pone.0176573.s004.pdf]

S3 Table. The table lists the general statistics of the proteobacterial taxa examined in this work.

class	all considered	percentage of total genera#	present in only one microbiome	present in at least two microbiomes	present in at least three microbiomes
**alpha** proteobacteria	190	36.8%	54	137	112
**beta** proteobacteria	84	37.3%	10	74	62
**gamma** proteobacteria	147	26.8%	45	102	79
**delta** proteobacteria	58	49.6%	15	43	32
**epsilon** proteobacteria	10	37.0%	0	10	8
total	**489**	34.1%	115	364	293

#per class, from NCBI taxonomy webpage:
http://www.ncbi.nlm.nih.gov/Taxonomy/Browser/wwwtax.cgi?id=1224 , accessed 5 September 2016

References:

1. Staudacher H, Kaltenpoth M, Breeuwer JA, Menken SB, Heckel DG, Groot AT. Variability of bacterial communities in the moth Heliothis virescens indicates transient association with the host. PLoS One. 2016;11(5): e0154514. doi: 10.1371/journal.pone.0154514
2. Halpern M, Senderovich Y. Chironomid microbiome. Microb Ecol. 2015;70(1): 1-8. doi: 10.1007/s00248-014-0536-9
3. Sharma P, Sharma S, Maurya RK, Das De T, Thomas T, Lata S, et al. Salivary glands harbor more diverse microbial communities than gut in Anopheles culicifacies. Parasit Vectors. 2014;7:235. doi: 10.1186/1756-3305-7-235
4. Dillon RJ, Webster G, Weightman AJ, Keith Charnley A. Diversity of gut microbiota increases with aging and starvation in the desert locust. Antonie Van Leeuwenhoek. 2010;97(1): 69-77.
